# The spliceosome, a potential Achilles heel of MYC-driven tumors

**DOI:** 10.1186/s13073-015-0234-3

**Published:** 2015-10-22

**Authors:** Olga Anczuków, Adrian R. Krainer

**Affiliations:** Cold Spring Harbor Laboratory, Bungtown Road, Cold Spring Harbor, NY 11724 USA

## Abstract

Alterations in RNA splicing are frequent in human tumors. Two recent studies of lymphoma and breast cancer have identified components of the spliceosome — the core splicing machinery — that are essential for malignant transformation driven by the transcription factor MYC. These findings provide a direct link between MYC and RNA splicing deregulation, and raise the exciting possibility of targeting spliceosome components in MYC-driven tumors.

## Spliceosome alterations in cancer

We have known for many years that human tumors exhibit abnormal splicing patterns. But in the past few years, we have started to appreciate that many of these changes reflect alterations in particular components of the splicing machinery. The core spliceosome (and associated regulatory factors) comprises more than 300 proteins and five small nuclear RNAs (snRNAs), and catalyzes both constitutive and regulated alternative splicing [1]. The U1, U2, U4, U5, and U6 snRNAs participate in several key RNA–RNA and RNA–protein interactions during spliceosome assembly and splicing catalysis. These snRNAs associate with seven ‘Sm’ core proteins and additional proteins to form small nuclear ribonucleoprotein (snRNP) particles. Other protein subcomplexes, such as the SF3A and SF3B complexes as well as the PRP19-associated complexes dubbed NTC and NTR, also play key roles in RNA splicing. The architecture of the spliceosome undergoes extensive remodeling in preparation for, during, and after splicing.

Recently, large-scale sequencing projects have identified recurrent somatic mutations in certain components of the spliceosome, such as SF3B1, U2AF1, SRSF2 and ZRSR2, in several types of hematological malignancies, including myelodysplastic syndromes (MDS), other myeloid neoplasms, and chronic lymphocytic leukemia (reviewed in [2]). The mutations that affect SRSF2 or U2AF1 directly impair hematopoietic differentiation in vivo, and result in changes in mRNA splicing patterns. Interestingly, in the case of SRSF2, the mutant protein exhibits altered RNA-binding specificity, rather than a loss of RNA-binding activity [2].

In addition, changes in splicing factor levels are frequently present in solid tumors. Several regulatory splicing factors, such as SRSF1, SRSF6, HNRNPA2/B1 or HNRNPH, have oncogenic properties, whereas others, including RBM5, RBM6 or RBM10, act as tumor suppressors (reviewed in [3]). These RNA-binding proteins elicit changes in alternative splicing in a concentration-dependent manner, and, thus, changes in their levels can alter the pre-mRNA splicing of many genes related to cancer, even in the absence of mutations. Alternative splicing has been linked to cancer through post-transcriptional regulation of components of many of the cellular processes considered to be ‘hallmarks’ of cancer, including cell proliferation, apoptosis, metabolism, invasion, and angiogenesis, but the biological consequences of these global changes in alternative splicing are only beginning to be unraveled.

Two recent studies [4, 5] have revealed that components of the spliceosome are essential for MYC (a transcription factor) to function as an oncoprotein. As *MYC* is the most frequently amplified oncogene in human cancers and plays a crucial role in transformation, therapies that exploit the spliceosome would be very attractive.

## MYC and alternative splicing in cancer

**Fig. 1:**
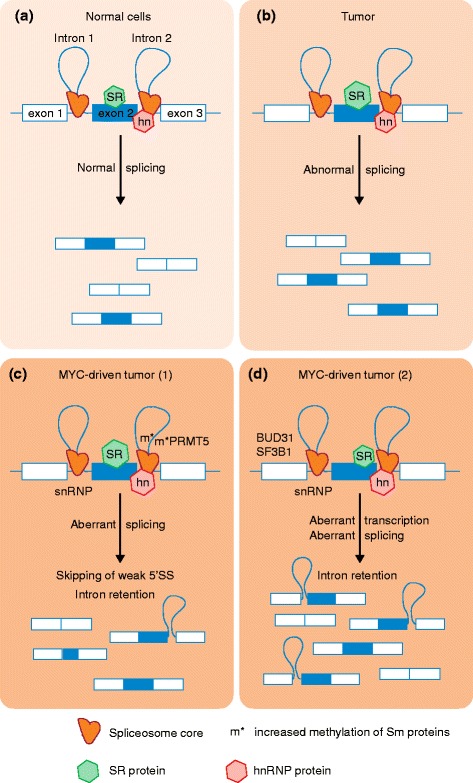
Splicing alterations in tumors. **a** In normal cells, the spliceosome, which is regulated by activators and repressors such as various serine-arginine-rich (*SR*) and heterogeneous nuclear ribonucleoprotein (*hn*) proteins, catalyzes pre-mRNA splicing, resulting in a normal, cell-type-specific splicing pattern. **b** In tumors, upregulation of certain splicing factors, for example SR proteins, or mutations in these factors promote abnormal splicing [3, 6, 7], leading to cancer-specific splicing patterns. **c** In the context of MYC-driven tumors, MYC directly upregulates transcription of splicing components, such as the splicing activator SR proteins and repressor hnRNP proteins [3, 6, 7], the PRMT5 methyltransferase, which controls Sm protein methylation [5], or the genes encoding snRNP constituents or snRNP assembly factors [5]. MYC-driven cancer cells exhibit aberrant splicing patterns, characterized by increased intron retention, and by increased skipping of exons that have weak 5′ splice sites (SS). **d** Alternatively, hyperactivation of MYC can lead to global upregulation of pre-mRNA levels, without directly affecting the expression of spliceosome components, and this excess of pre-mRNA overwhelms the splicing machinery [4]

Previous work linked MYC and alternative splicing by demonstrating that genes that encode certain splicing activators and repressors, such as *SRSF1*, *HNRNPA1*, *HNRNPA2* or *PTB*, are direct transcriptional targets of MYC [3, 6, 7]. Furthermore, *SRSF1* has been shown not only to contribute to MYC’s oncogenic activity [7] but also to cooperate with MYC in malignant transformation, promoting the formation of more-aggressive breast tumors [6]. The recent reports by Koh et al. [5] and Hsu et al. [4] have provided a direct link between MYC and the core splicing machinery by identifying components of the spliceosome that are essential for MYC’s role in transformation (Fig. 1).

By screening for genes whose downregulation is synthetic lethal in the presence of hyperactive MYC in human mammary epithelial cells, Hsu and colleagues identified at least five spliceosome components [4]: SF3B1 and U2AF1, two of the proteins frequently mutated in MDS; SNRPF, one of the Sm proteins of spliceosomal snRNPs; EFTUD2, a component of U5 snRNP; and BUD31, a protein associated with the PRP19-related or NTR complex [1]. Notably, BUD31 knockdown in the MYC hyperactivated state led to decreased cell viability and increased apoptosis, and was associated with the accumulation of transcripts with one or more retained introns. The authors observed a decrease in poly(A) + RNA after actinomycin D treatment, which they interpreted as symptomatic of a defect in pre-mRNA maturation and/or stability. Interestingly, BUD31 knockdown did not confer sensitivity to cells expressing human epidermal growth factor receptor 2 (HER2) or epidermal growth factor receptor (EGFR), demonstrating that limiting BUD31 is not synthetic lethal with all oncogenes. Finally, pharmacological inhibition of the core spliceosome component SF3B1 reduced the tumorigenic and metastatic potential of MYC-driven human breast cancer cell lines. The authors suggest that oncogenic MYC overloads the splicing machinery in mammary epithelial cells by increasing total transcript levels, making the cells more sensitive to perturbations in splicing fidelity.

In parallel work, Koh and colleagues identified several components of the splicing machinery as key effectors of MYC in lymphomagenesis in the Eμ-myc mouse model [5]. In this model, transgenic mice express the c-*myc* oncogene under the control of the IgM heavy-chain enhancer, and reproducibly develop and die from tumors of the B-lymphocyte lineage. These authors report that during lymphomagenesis, MYC directly upregulates transcription of genes encoding snRNP constituents or snRNP assembly factors, including: GEMIN5, a component of the SMN complex that loads a ring of seven Sm proteins onto snRNAs; the Sm proteins SNRPD1, SNRNPD3, and SNRNPB; the arginine methyltrantransferase PRMT5, which methylates arginines in the Sm proteins; and WDR77, a non-catalytic component of the ormethylosome, a methyltransferase complex. In addition, lymphoma development was delayed in Eμ-myc-PRMT5^+/−^ mice. PRMT5 depletion led to a reduction of Sm protein methylation, which was associated with the accumulation of retained introns and the skipping of alternative exons that have weak 5′ splice sites, and it resulted in increased apoptosis. Furthermore, by using antisense oligonucleotides, the authors demonstrate the contribution of several splicing events to the PRMT5^+/−^ phenotype in Eμ-myc B cells. These findings suggest that in B lymphocytes, oncogenic MYC reprograms the spliceosome to drive inclusion of alternative exons with weak 5′ splice sites.

Both studies uncovered an essential role of the splicing machinery in MYC-driven transformation, and identify multiple associated abnormal splicing events, including intron retention. Interestingly, widespread intron retention was recently described as a common event across human tumors, even in the absence of mutations that directly affect the spliceosome [8]. In addition, changes in intron processing have been reported during embryonic development, as well as during the response to DNA damage. These observations suggest that specific intron-retention events may be a signature of responses to various cell stresses. Interestingly, the mechanisms through which MYC appears to alter splicing in the context of lymphomagenesis differ from those in breast cancer. In the former context, MYC hyperactivation affects the levels of specific splicing regulators [5], whereas in the latter context, it promotes a global increase in pre-mRNA levels [4] (although upregulation of a splicing activator has been reported previously). These ostensibly different findings suggest that many of the splicing changes associated with cancer are likely to be context-dependent.

## Opportunities for therapeutic intervention

In light of these findings, both Hsu et al. and Koh et al. explored the therapeutic potential of targeting splicing in MYC-driven tumors. The idea of targeting the spliceosome is not new, and the first spliceosome inhibitory compounds were initially identified in the late 1990s, while characterizing anti-tumor drugs. However, recent improvements in chemistry, as well as a better understanding of the modes of action of these molecules, have created novel therapeutic opportunities (reviewed in [9]). Hsu et al. demonstrate that genetic knockdown of BUD31 or SF3B1, or pharmacological inhibition of SF3B1, can delay both primary tumor onset and metastasis formation following injection of MYC-expressing human breast cancer cells lines in mice [4]. Similarly, Koh et al. [5] demonstrate that PRMT5 haploinsufficiency delays MYC-driven lymphomagenesis in mice. Thus, both studies suggest that a therapeutic window for splicing inhibition exists in MYC-driven cancers.

Two conceptually different approaches to splicing inhibition are currently being tested. The first targets general components of the splicing machinery and inhibits splicing at a global level, for example using small-molecule inhibitors to target the SF3B complex or the kinases that phosphorylate SR proteins [9]. These drugs inhibit very basic steps in splice site recognition, and potentially have broad cytotoxic effects. Nevertheless, several studies have reported that cancer cells are more sensitive to these drugs than normal cells [9], suggesting that general inhibition of splicing could be a viable anti-tumor strategy. The second approach is to target a tumor-specific splicing event directly, for example by using antisense oligonucleotides that bind to a transcript in a sequence-specific manner to redirect splicing (reviewed in [10]). This approach is expected to have fewer off-target effects and might be more tumor-specific. Identifying a key splicing event, or more likely a set of splicing events, that is required for transformation and tumor maintenance will, however, require a systematic effort.

Although *MYC* is the most frequently amplified oncogene in human cancers and plays a crucial role in transformation, therapeutic strategies that target MYC-driven tumors are very limited at present. Thus, targeting either the spliceosome or specific splicing events could potentially provide novel therapeutic targets in the context of MYC-driven tumors. A detailed understanding of the cell-type-specific mechanisms through which splicing contributes to transformation in cooperation with *MYC* (but not with other oncogenes) should facilitate the translation of the new findings to the clinic.

## Abbreviations

MDS: myelodysplastic syndromes
snRNA: small nuclear RNA
snRNP: small nuclear ribonucleoprotein particle
